# Bloodstream Infections Caused by *Magnusiomyces capitatus* and *Magnusiomyces clavatus*: Epidemiological, Clinical, and Microbiological Features of Two Emerging Yeast Species

**DOI:** 10.1128/aac.01834-21

**Published:** 2022-02-15

**Authors:** Janina Noster, Martin B. Koeppel, Marie Desnos-Olivier, Maria Aigner, Oliver Bader, Karl Dichtl, Stephan Göttig, Andrea Haas, Oliver Kurzai, Arthur B. Pranada, Yvonne Stelzer, Grit Walther, Axel Hamprecht

**Affiliations:** a Carl von Ossietzky University Oldenburg, Department of Medical Microbiology and Virology, Oldenburg, Germany; b Max-von-Pettenkofer Institute, Faculty of Medicine, LMU Munich, Munich, Germany; c Institut Pasteur, CNRS, Molecular Mycology Unit, National Reference Center for Invasive Mycoses & Antifungals, UMR2000, Paris, France; d Medical University Innsbruck, Division of Hygiene and Medical Microbiology, Innsbruck, Austria; e Institute for Medical Microbiology and Virology, University Medical Center Göttingen, Göttingen, Germany; f Institute for Medical Microbiology and Infection Control, Hospital of Johann Wolfgang Goethe University, Frankfurt, Germany; g University of Würzburg, Institute for Hygiene and Microbiology, Würzburg, Germany; h German National Reference Centre for Invasive Fungal Infections, Leibniz Institute for Natural Product Research and Infection Biology—Hans Knöll Institute, Jena, Germany; i Medical Center Dr. Eberhard & Partner Dortmund (ÜBAG), Department of Microbiology, Dortmund, Germany; j German Centre for Infection Research, Partner Site Bonn-Cologne, Cologne, Germany; k University of Cologne, University Hospital, Institute for Medical Microbiology, Immunology and Hygiene, Cologne, Germany

**Keywords:** *Saprochaete clavata*, *Saprochaete capitata*, *Magnusiomyces capitatus*, *Magnusiomyces clavatus*, *Geotrichum*, bloodstream infection, MIC

## Abstract

Magnusiomyces clavatus and Magnusiomyces capitatus are emerging yeasts with intrinsic resistance to many commonly used antifungal agents. Identification is difficult, and determination of susceptibility patterns with commercial and reference methods is equally challenging. For this reason, few data on invasive infections by *Magnusiomyces* spp. are available. Our objectives were to determine the epidemiology and susceptibility of *Magnusiomyces* isolates from bloodstream infections (BSI) isolated in Germany and Austria from 2001 to 2020. In seven institutions, a total of 34 *Magnusiomyces* BSI were identified. Identification was done by internal transcribed spacer (ITS) sequencing and matrix-assisted laser desorption ionization–time of flight mass spectrometry (MALDI-TOF MS). Antifungal susceptibility was determined by EUCAST broth microdilution and gradient tests. Of the 34 isolates, *M. clavatus* was more common (*n* = 24) than *M. capitatus* (*n* = 10). BSI by *Magnusiomyces* spp. were more common in men (62%) and mostly occurred in patients with hemato-oncological malignancies (79%). The highest *in vitro* antifungal activity against *M. clavatus*/*M. capitatus* was observed for voriconazole (MIC_50_, 0.03/0.125 mg/L), followed by posaconazole (MIC_50_, 0.125/0.25 mg/L). *M. clavatus* isolates showed overall lower MICs than *M. capitatus*. With the exception of amphotericin B, low essential agreement between gradient test and microdilution was recorded for all antifungals (0 to 70%). Both species showed distinct morphologic traits on ChromAgar Orientation medium and Columbia blood agar, which can be used for differentiation if no MALDI-TOF MS or molecular identification is available. In conclusion, most BSI were caused by *M. clavatus.* The lowest MICs were recorded for voriconazole. Gradient tests demonstrated unacceptably low agreement and should preferably not be used for susceptibility testing of *Magnusiomyces* spp.

## INTRODUCTION

The closely related species Magnusiomyces capitatus and Magnusiomyces clavatus are ascomycetous yeasts known to cause life-threatening invasive infections in immunocompromised patients, particularly in the case of hemato-oncological malignancies (1–4). Both species have undergone frequent taxonomic changes: *M. capitatus* was formerly known as Saprochaete capitata, Geotrichum capitatum, Blastoschizomyces capitatus, Trichosporon capitatum, or Dipodascus capitatus and *M. clavatus* as Saprochaete clavata or Geotrichum clavatum (5).

Infections with these species are associated with a high mortality rate of 40 to 80% (1, 6–8). In recent years, the numbers of published cases of infections with *M. clavatus* and *M. capitatus* have increased, and both species are considered emerging pathogens (4, 8–10). Additionally, outbreaks in hospitals have been described, the largest one in 2013 in France with 39 cases (4).

Rising infection rates may be due to an increasing number of patients at risk and the frequent use of echinocandins, which may predispose to infections with *Magnusiomyces* spp. due to intrinsic resistance against these antifungal agents (11, 12). Despite its clinical relevance, clinical and susceptibility data on *M. clavatus*/*capitatus* are still scarce and mostly originate from case reports or small case series, often without reference methods being used for identification and susceptibility testing. The correct identification of both species is challenging, and *M. clavatus* is frequently misidentified as *M. capitatus* (13, 14) by biochemical identification methods, but may also be misclassified by matrix-assisted laser desorption ionization–time of flight mass spectrometry (MALDI-TOF MS) and molecular identification methods (15, 16), depending on the system and database used. These erroneous results may skew epidemiological studies and likely lead to an underestimation of *M. clavatus* infections. Only a few data on susceptibility of *Magnusiomyces* spp. to antifungal agents are available, and even fewer have been obtained using the reference method broth microdilution (BMD) (6, 17–19).

In the present study, we aimed to analyze the epidemiology and susceptibility of *Magnusiomyces* infections on a larger scale. *Magnusiomyces* isolates obtained from bloodstream infections (BSI) at seven study centers in the years 2001 to 2020 were retrospectively analyzed.

## RESULTS

### Identification of *Magnusiomyces clavatus* and *Magnusiomyces capitatus* isolates.

Thirty-four nonduplicate isolates from patients with bloodstream infections were available for analysis. Of these, 10 were *M. capitatus* and 24 *M. clavatus*, as identified by internal transcribed spacer (ITS) sequencing (see Table S1 in the supplemental material).

Since identification of pathogens on the molecular level is not feasible in the daily lab routine, all isolates were additionally analyzed using MALDI-TOF MS. Using the time-saving direct transfer protocol, 15/24 *M. clavatus* and 7/10 *M. capitatus* isolates (65%) were successfully identified using the MALDI Biotyper system, with quality scores reaching from 1.74 to 2.23. Tube-based extraction finally allowed successful identification for 100% of all tested isolates, with scores ranging from 1.79 to 2.26 (Table S1).

### Morphology of *Magnusiomyces* isolates on different agars.

**FIG 1:**
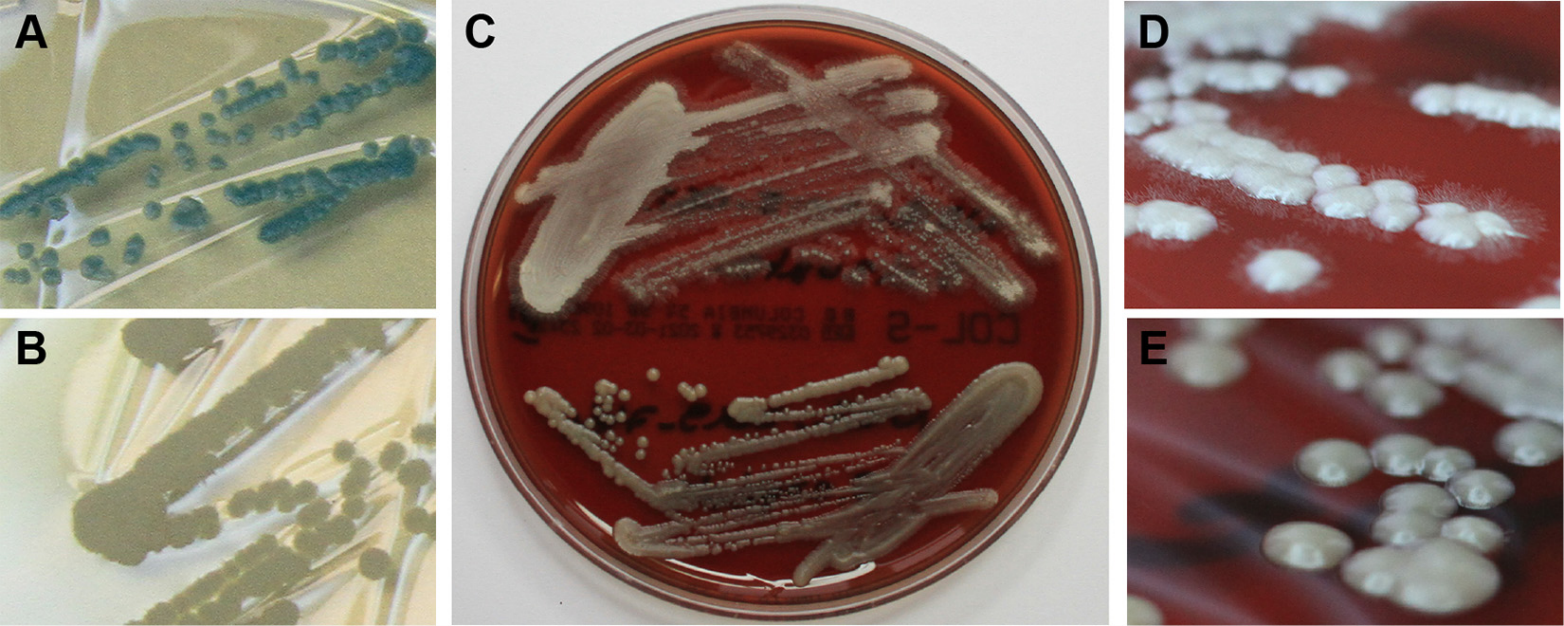
*M. clavatus* and *M. capitatus* colony morphology. (A and B) Morphology of *M. clavatus* (A) and *M. capitatus* (B) on Orientation medium (ChromAgar). (C) Morphology on Columbia blood agar (CBA), with *M. clavatus* in the upper half and *M. capitatus* in the lower half. On CBA, most isolates of *M. clavatus* showed colonies with filamentous margins (D), in contrast to *M. capitatus* (E).

Macroscopic morphology of all isolates was assessed on a total of seven different agars. On most agars, there was little difference between *M. capitatus* and *M. clavatus*. The most obvious difference was observed with CHROMagar Orientation medium (ORI medium): While all *M. capitatus* isolates grew in whitish colonies (10/10), most *M. clavatus* isolates showed a greenish-blue appearance after 24 h of incubation (23/24 isolates [96%]) on this agar (Fig. 1A and B and Table 1), which increased to 100% (24/24) after 48 h. Comparable differences were observed using chromID CPS Elite medium (CPS), but color differentiation was less distinct. Additionally, when grown on Columbia blood agar (CBA) for 48 to 72 h, *M. capitatus* grew in rather smooth colonies, often with a yellowish color and an only slightly filamentous colony border (9/10 [90%]). In contrast, colonies of *M. clavatus* were white and had a more filamentous colony morphology (19/24 [79%]) (Fig. 1C, D, and E and Table 1).

**TABLE 1:** Distribution of colony morphological characteristics of *M. clavatus* and *M. capitatus* among the tested isolates after growth on CPS Elite agar, Orientation agar, and Columbia blood agar^*a*^

Species	No. (%) of colonies grown on:						
	CPS			ORI		CBA	
	Green	Brown	White	Blue	White	Filamentous	Slightly filamentous/smooth
*M. clavatus* (*n* = 24)	22 (92)	1 (4)	1 (4)	24 (100)	0 (0)	19 (79)	5 (21)
*M. capitatus* (*n* = 10)	0 (0)	9 (90)	1 (10)	0 (0)	10 (100)	1 (10)	9 (90)

a CPS, CPS Elite agar; ORI, Orientation agar; CBA, Columbia blood agar.

### MIC determination for *Magnusiomyces* isolates by broth microdilution.

EUCAST recommends RPMI 1640 medium and an inoculum of 0.5 McFarland standard (McF) for susceptibility testing of yeasts. However, when viable colony counts for *Magnusiomyces* spp. were determined using these parameters, colony counts were below 0.5 × 10^5^ to 2.5 × 10^5^ CFU/mL, as required by EUCAST (20). Equally, the absorbance at 24 h was below 0.2, demonstrating poor growth in RPMI 1640. Therefore, a suspension equivalent to 0.75 McF was used to obtain viable colony counts within the predefined range, and reading was performed at 48 h, which allowed reproducible determination of susceptibility. The lowest MICs for *M. clavatus*/*M. capitatus* were recorded for voriconazole (MIC_50_, 0.03/0.125 mg/L), posaconazole (MIC_50_, 0.125/0.25 mg/L), and itraconazole (MIC_50_, 0.25/0.5 mg/L), whereas higher MICs were observed for amphotericin B (MIC_50_, 1/1 mg/L), fluconazole (MIC_50_, 2/2 mg/L), and caspofungin (MIC_50_, 2/>4 mg/L) (Table 2; see Fig. S1 in the supplemental material).

**TABLE 2:** Antifungal MICs by broth microdilution of *M. clavatus* and *M. capitatus* isolates^*a*^

Antifungal	Species	Total	No. of isolates with MIC (mg/L)															MIC (mg/L)		
			0.0039	0.0078	0.0156	0.0313	0.0625	0.125	0.25	0.5	1	2	>2	4	>4	8	16	MIC_50_	MIC_90_	Range
AMB	*M. clavatus*	24				0	1	0	0	6	17	0		0		0	0	1	1	0.0625 to 1
	*M. capitatus*	10				0	0	0	0	3	5	2		0		0	0	1	2	0.5 to 2

FLC	*M. clavatus*	24							1	4	2	10		4		1	2	2	8	0.25 to 16
	*M. capitatus*	10							2	1	1	2		0		3	1	2	8	0.25 to 16

ITC	*M. clavatus*	24	0	2	1	3	1	1	10	4	1	0		1				0.25	0.5	0.0078 to 4
	*M. capitatus*	10	0	0	0	1	1	0	1	4	1	2		0				0.5	2	0.03 to 2

VRC	*M. clavatus*	24	0	6	2	7	5	2	1	1	0	0		0		0		0.03	0.125	0.0078 to 0.5
	*M. capitatus*	10	0	0	0	2	1	3	3	1	0	0		0		0		0.125	0.25	0.03 to 0.5

POS	*M. clavatus*	24	1	3	2	2	1	8	5	1	0	0		1		0		0.125	0.25	0.0039 to 4
	*M. capitatus*	10	0	0	2	0	0	0	4	2	0	2		0		0		0.25	2	0.0156 to 2

AFG	*M. clavatus*	24			0	0	0	0	1	7	11	3		1	1	0		1	2	0.25 to >4
	*M. capitatus*	10			0	0	0	0	0	4	1	2		2	1	0		1	4	0.5 to >4

CAS	*M. clavatus*	24			0	0	0	0	0	1	8	5		1	9	0		2	>4	0.5 to >4
	*M. capitatus*	10			0	0	0	0	0	0	0	0		1	9	0		>4	>4	4 to >4

MFG	*M. clavatus*	24			0	0	0	1	3	9	2	3	6	0		0		0.5	>2	0.125 to >2
	*M. capitatus*	10			0	0	0	0	0	0	0	0	10	0		0		>2	>2	>2

a AMB, amphotericin B; FLC, fluconazole; ITC, itraconazole; VRC, voriconazole; POS, posaconazole; AFG, anidulafungin; CAS, caspofungin; MFG, micafungin.

### MIC determination for *Magnusiomyces* isolates by gradient tests.

Since microdilution testing is unavailable in most routine laboratories, gradient tests are usually used for susceptibility testing of rare yeasts due to ease of handling and reduced hands-on time. To evaluate the accuracy of gradient tests compared to the microdilution method, the essential agreement (EA) was determined for amphotericin B, itraconazole, voriconazole, and fluconazole (see Table S2 and Fig. S2 in the supplemental material) at 24 and 48 h. While for amphotericin B, an essential agreement of 92% was recorded for *M. clavatus* isolates, only 80% was observed for *M. capitatus* isolates, regardless of the reading time. For all other antifungal agents, the EA ranged from 13 to 71% for *M. clavatus* to 0 to 50% for *M. capitatus*. For voriconazole, the EA was better after 24 h for *M. capitatus* and *M. clavatus*. The lowest EA was determined for fluconazole at 48 h. For this antifungal agent, MIC values differed by more than one dilution in 88% and even 100% of isolates for *M. clavatus* and *M. capitatus*, respectively.

### Molecular analysis of *M. clavatus* isolates.

Based on whole-genome sequencing, Vaux et al. defined two clades (A and B) for the *M. clavatus* isolates of the 2012 outbreak in France (4). These clades could be separated by eight single nucleotide polymorphisms (SNPs). After PCR and sequencing of these SNPs, none of the German *M. clavatus* isolates belonged to either clade A or clade B. Furthermore, concatenate sequences of the eight regions (corresponding to 5,968 bp) of the 24 German isolates and the ex-type strain CBS 425.71 have 100% similarity, confirming the highly conserved genome of *M. clavatus*. For *M. capitatus*, no typing scheme is currently available.

### Clinical data.

For 33/34 patients, clinical information was available. Of these, 20 (59%) were male; age ranged from 4 to 78 years (median, 57 years). The majority (26/33 [79%]) were patients with a hemato-oncological disease. Acute myeloid leukemia (AML) was overall the most common diagnosis (*n* = 12), followed by acute lymphatic leukemia (ALL) (*n* = 6), chronic lymphocytic leukemia (CLL), and Hodgkin’s lymphoma and anaplastic T-cell leukemia (*n* = 1 each) (Table 3). Two bloodstream infections were recorded in patients with solid tumors. Blood cultures (BC) became positive between day 0 (the day BC was taken) and day 5 (median, day 2). The duration of fungemia ranged from 1 to 17 days (median, 6 days) and persisted until death in four patients. Information on the focus of infection was available in 19 patients: 11 (58%) had a fungal pneumonia or presumed respiratory focus, five patients (26%) had a catheter-related infection, and in three patients (16%) a gastrointestinal focus was identified, while in 16 patients, the focus of infection was unknown or no data were available. In addition to the positive blood culture, *Magnusiomyces* was detected in other clinical specimen in 23 patients; of these respiratory samples (bronchoalveolar lavage [BAL] fluid, sputum, or throat swab) were positive in nine patients, gastrointestinal samples in 14 patients (stool, *n* = 9; abdominal swab, *n* = 2; other, *n* = 3), and urine cultures in two patients. For the remaining 10 patients, other samples were negative or not taken. Neutropenia was recorded in 20 patients at the time the blood culture was drawn, and six patients were not neutropenic, while for seven patients, no information on leukocyte count was available. Amphotericin B was the most commonly used antifungal (*n* = 10), followed by voriconazole (*n* = 7), fluconazole (*n* = 3), and caspofungin (*n* = 3) (Table 3). Crude mortality was 50% (13/26) at day 30 and 66.7% (16/24) at day 100.

**TABLE 3:** Clinical characteristics of patients with *Magnusiomyces* BSI^*a*^

Patient	Yr	Duration of fungemia (days)	Focus of BSI	Other specimen(s) with *Magnusiomyces* spp.	Hemato-oncology patient	Malignancy	Neutropenia	Therapy	Survival	
									Day 30	Day 100
*M. clavatus* (*n* = 24)										
1	2001	9	Unknown	−	Yes	Aplastic anaemia	NA	AMB	Alive	Alive
2	2003	6	Fungal pneumonia	Urine, respiratory	Yes	AML	Yes	AMB, VRC, 5FC	Deceased	Deceased
3	2003	No follow-up	Fungal pneumonia	Stool	Yes	AML	Yes	NA	Deceased	Deceased
4	2004	No follow-up	Unknown	Knee aspirate	Yes	Unknown	NA	NA	Alive	Alive
5	2004	No follow-up	Unknown	−	Yes	AML	Yes	NA	Deceased	Deceased
6	2005	NA	Unknown	Gall bladder	Yes	Gall bladder tumor	NA	NA	NA	NA
7	2006	NA	Unknown	Spleen biopsy	Yes	Previous BM transplantation	Yes	AMB + FLC	Deceased	Deceased
8	2006	NA	Unknown	Subphrenical aspirate	No	−	NA	FLC	NA	NA
9	2007	2	Fungal pneumonia	Respiratory	Yes	AML	Yes	CAS	Alive	Alive
10	2008	Persisting	Gastrointestinal	Stool	Yes	AML	Yes	NA	Deceased	Deceased
11	2009	4	Unknown	−	Yes	AML	Yes	VRC	Alive	Deceased
12	2010	3	Fungal pneumonia	Stool, groin swab, throat swab	Yes	AML	Yes	NA	Alive	Alive
13	2010	17	Catheter	Hickman line	Yes	AML+ MDS	Yes	AMB, FLC	Alive	Alive
14	2011	No follow-up	Unknown	Respiratory	No	−	No	NA	Alive	NA
15	2011	10	Catheter, abdominal	Abdominal swab	No	−	No	NA	Alive	Alive
16	2011	No follow-up	Fungal pneumonia	−	Yes	AML	Yes	AMB, VRC	Deceased	Deceased
17	2012	11	Infection port a catheter	Stool	Yes	ALL	Yes	AMB, VRC	Deceased	Deceased
18	2012	≥3, positive again after d57	Unknown	Stool, respiratory, pericardial effusion	Yes	Large cell anaplastic T-cell lymphoma	Yes	AMB, VRC	Alive	Deceased
19	2012	Persisting	Fungal pneumonia	Stool	Yes	ALL	Yes	NA	Deceased	Deceased
20	2013	Persisting	Fungal pneumonia		Yes	AML	Yes	AMB, VRC	Deceased	Deceased
21	2013	Persisting	Fungal pneumonia	Stool, respiratory	Yes	ALL	Yes	AMB, CAS	Deceased	Deceased
22	2013	6	Unknown	Stool	Yes	ALL	NA	MFG, AMB	Deceased	Deceased
23	2015	1	NA	−	No	−	No	NA	NA	NA
24	2020	6	Fungal pneumonia	Urine, stool, throat swab	Yes	CLL	Yes	NA	Alive	NA
Total	2001 to 2020				20 yes, 4 no	10 AML, 4 ALL, 5 others, 5 unknown/no malignancy	16 yes, 3 no, 5 NA	10 AMB, 6 VRC, 3 FLC, 2 CAS, 1 MFG, 1 5FC, 11 NA	10 alive, 11 deceased, 3 NA	6 alive, 13 deceased, 5 NA

*M. capitatus* (*n* = 10)										
25	2001	5	Respiratory + abdominal	Respiratory, abdominal swab	No	−	No	NA	Deceased	Deceased
26	2004	NA	Unknown	NA	Yes	Unspecified	Yes	NA	NA	NA
27	2007	8	Unknown	−	Yes	Hodgkin lymphoma, secondary MDS	Yes	VRC, CAS	Deceased	Deceased
28	2008	13	Wound, intestine	Anal wound	Yes	ALL	Yes	NA	Alive	Deceased
29	2009	4	Fungal pneumonia	Throat swab	Yes	AML	Yes	NA	Alive	Alive
30	2013	No follow-up	Catheter	Central line	Yes	ALL	No	NA	NA	NA
31	2015	NA	NA	NA	NA	NA	NA	NA	NA	NA
32	2016	1	Catheter	Central line	No	−	No	NA	Alive	Alive
33	2019	NA	NA	NA	Yes	Astrocytoma	NA	NA	NA	NA
34	2020	NA	NA	NA	Yes	AML	NA	NA	NA	NA
Total	2001 to 2020				7 yes, 2 no, 1 NA	2 AML, 2 ALL, 2 other, 1 unspecified, 3 unknown/no malignancy	4 yes, 3 no, 3 NA	1 VRC, 1 CAS, 9 NA	3 alive, 2 deceased, 5 NA	2 alive, 3 deceased, 5 NA

a BSI, bloodstream infections; AML, acute myeloid leukemia; ALL, acute lymphatic leukemia; CLL, chronic lymphocytic leukemia; MDS, myelodysplastic syndrome; −, not present; NA, not available; AMB, amphotericin B; FLC, fluconazole; VRC, voriconazole; CAS, caspofungin; MFG, micafungin; 5FC, 5-flucytosin.

## DISCUSSION

In the present study, most bloodstream infections (24/34 [71%]) were caused by *M. clavatus*, in contrast to previous publications, which mainly report on *M. capitatus* (1, 6, 21–25). However, this could be caused by misidentification of *M. clavatus* as *M. capitatus*, which can occur with biochemical methods. These have mainly been used in older studies, but MALDI-TOF MS and molecular methods can also lead to erroneous identifications (14, 15). In the present study, species identification by MALDI-TOF MS using the MALDI Biotyper was 100% concordant with ITS sequencing data. Contrarily, previous studies reported frequent failure upon use of the VitekMS MALDI-TOF system using an older database (prior to version 3.2) (14, 15), with the exception of a recent study from China (26).

Interestingly, the two species showed distinctive morphologies on ORI medium and Columbia blood agar, which could be useful for the preliminary differentiation of *Magnusiomyces* isolates or in a low-resource setting: e.g., when MALDI-TOF MS or sequencing is not available. ORI is a chromogenic agar usually used for the differentiation of *Enterobacterales*. The greenish-blue color observed for *M. clavatus* on ORI medium indicates β-glucosidase activity, which appears to be absent, or strongly reduced, in *M. capitatus*.

Besides identification, susceptibility testing of *Magnusiomyces* species isolates is challenging. EUCAST broth microdilution has been developed for fast-growing yeasts like *Candida* spp. Limited growth of *Magnusiomyces* isolates in RPMI 1640 medium complicating MIC determination has previously been reported (27). Additionally, we observed that for *Magnusiomyes* spp., a suspension equivalent to 0.5 McF does not correspond to the viable inoculum count required by EUCAST. Our results suggest that the yeast suspension should be adjusted to 0.75 McF and viable colony counts should be determined in order to ascertain the desired inoculum; in addition, the spectrophotometric readout should be performed at 48 h.

Overall, the lowest MICs were recorded for voriconazole (MIC_50_, 0.03/0.125 mg/L for *M. clavatus*/*M. capitatus*), followed by posaconazole and itraconazole. MICs for amphotericin B were higher (MIC_50_, 1/1 mg/L for *M. clavatus*/*M. capitatus*). As currently no clinical breakpoints are available for *Magnusiomyces* spp., the activity of these antifungals cannot be well compared. *M. capitatus* isolates overall showed higher MICs for azoles and amphotericin B compared to *M. clavatus* (Table 2; Fig. S1). MICs of azoles were comparable to data from previous studies (6, 8, 9, 28, 29), and MICs of anidulafungin and caspofungin were high, as anticipated (9, 30). In contrast, MICs of micafungin were lower, with *M. clavatus* isolates having significantly lower MICs (MIC_50_, 0.5 mg/L) compared to *M. capitatus* (MIC_50_, 2 mg/L; *P* < 0.001). Whether this observation and the overall lower MICs of *M. clavatus* for other antifungals indicate a species-specific difference needs to be investigated in future studies with higher isolate numbers.

Intrinsic resistance against fluconazole has been commonly reported for *M. capitatus* (7, 21). However, in the present study, 6/10 *M. capitatus* isolates and 17/24 *M. clavatus* isolates had fluconazole MICs of ≤2 mg/L, which is the breakpoint set by EUCAST for most *Candida* species. MIC_50_/MIC_90_ values were 2/8 mg/L for both *M. clavatus* and *M. capitatus*, which contrasts with the studies of Kaplan et al. (29) and Esposto et al. (6), who reported higher MICs when using the CLSI method or EUCAST and CLSI methods, respectively.

Given the low numbers of severe infections, treatment is currently based on extrapolated data from retrospective case series and expert opinion. Amphotericin B with or without flucytosine or voriconazole is mostly recommended for invasive infections (31), while caspofungin and fluconazole are avoided. Given the limited clinical information available for our cases and the presence of multiple confounders (e.g., neutropenia, different foci of infection, frequent combination therapy), the effect of the different antifungals on outcome cannot be reliably compared. Interestingly, one patient with *M. clavatus* BSI and a respiratory focus was treated successfully with caspofungin (MIC, 1 mg/L) (Table 3).

While BMD is the “gold standard” for MIC determination and primarily used in reference laboratories, agar gradient tests are frequently employed in routine laboratories due to ease of handling. The reliability of gradient tests was assessed for amphotericin B, itraconazole, voriconazole, and fluconazole. Overall, only amphotericin B showed acceptable essential agreement for *M. clavatus* (92%), which was lower for *M. capitatus* (80%). For all other antifungals, EA percentages were unacceptably low (0 to 71%, regardless of the species tested). If only MIC test strips are available, we recommend reading at 24 h for amphotericin B, voriconazole, and fluconazole, while itraconazole should be read at 48 h. Agreement of a different gradient test (Etest; bioMérieux) was on average higher when compared to CLSI microdilution in the study by Girmenia et al. (32). In contrast, higher MICs by Etest compared to BMD (Sensititre YeastOne) were observed for azoles in the study by Buchta et al. (9), similarly to the present study.

Most *Magnusiomyces* infections are reported from Mediterranean Europe (1, 24), but case numbers are also increasing in Central Europe (33), possibly due to global warming (34). The isolates examined in this study were from individual cases in Germany and Austria, and outbreaks of both species have not yet been reported in either country, but have been registered in neighboring countries, such as France, Switzerland, and Italy (7, 33). The German *M. clavatus* isolates were analyzed for relatedness to the outbreak strain using SNP analyses, but could not be assigned to either clade A or clade B reported from France, indicating independent emergence of *M. clavatus* infections in Germany. In 2014, Vaux et al. (4) also sequenced SNP regions for clinical and environmental isolates from other countries recovered between 1971 and 2010. None of these isolates belonged to clade A or clade B, suggesting that both clades could have a specific French origin. Based on whole-genome sequencing of 18 isolates, the authors also concluded that *M. clavatus* is a genetically highly monomorphic species since only 312 high-quality SNPs were discovered over the entire genome. This could be the result of a recent emergence or of a very low rate of mutation due to low environmental pressure, for example, but the natural reservoir of *M. clavatus* is still unknown.

This study has some limitations—mainly its retrospective character. Isolates were retrieved from strain collections at seven different institutions since 2000, and not all isolates from the respective strain collection were still available for analysis, which could have influenced the overall epidemiology. Additionally, only limited clinical information was available in some cases, and no analyses regarding treatment, outcome, or other risk factors could be performed. Nevertheless, the present multicenter study is one of the most comprehensive on *Magnusiomyces* bloodstream infections worldwide. Besides the large number of clinical isolates, reference methodology for identification and susceptibility testing was used, and techniques for identification and susceptibility in the routine laboratory were systematically assessed and improved.

### Conclusion.

In summary, we have demonstrated that more bloodstream infections in Germany were caused by *M. clavatus* than *M. capitatus*, and they occurred most commonly in patients with acute leukemia. Identification by MALDI Biotyper was reliable. Species differentiation of *M. capitatus* and *M. clavatus* can additionally be achieved by using morphological characteristics on chromogenic media and Columbia blood agar, which are widely available and do not require specialized equipment. MIC determination for *Magnusiomyces* spp. should be performed by broth microdilution using an inoculum of 0.75 McFarland standard. The lowest MICs among all antifungals were recorded for voriconazole, and *M. clavatus* isolates showed overall lower MICs than those for *M. capitatus*.

## MATERIALS AND METHODS

### Isolate collection and patient data.

Isolates from 34 different patients with bloodstream infections in the years 2001 to 2020 were retrieved from the laboratory databases of seven participating institutions in Germany and Austria. All isolates had previously been identified in the respective institutions as *M. capitatus*/*M. clavatus*, *Blastoschizomyces*, or *Geotrichum* species by different methods, including microscopy, biochemical analysis (Vitek2, API 32C [bioMérieux, Nürtingen, Germany]), or MALDI-TOF MS. Further analysis was performed at the central study site (i.e., the microbiology laboratory at the University of Cologne).

### Ethics.

The study was conducted in accordance with the Declaration of Helsinki and approved by the ethics committee of the University of Cologne (14-385). Patient data were obtained from the laboratory information systems and patients’ charts and documented in an anonymized form. The requirement for written informed consent was waived due to the observational, retrospective nature of this study.

### Identification and susceptibility testing.

Identification was carried out by MALDI Biotyper analysis (Compass software 4.1, Bruker Daltonics, Bremen, Germany) and sequencing of the internal transcribed spacer (ITS), as previously described (15). PCR and sequencing of the internal transcribed spacer (ITS) region of ribosomal DNA (rDNA) gene cluster were performed as recommended by CLSI (35). Obtained ITS sequences were analyzed using the curated database of MycoBank (https://www.mycobank.org/page/Pairwise_alignment) and additionally GenBank (https://blast.ncbi.nlm.nih.gov/Blast.cgi). For MALDI-TOF MS, samples were prepared using the time-saving direct transfer protocol and thresholds, as validated before (15). When no identification or low-confidence scores (≤1.7) were observed by direct transfer protocol, tube-based extraction was performed using 75% ethanol, formic acid, and acetonitrile.

Susceptibility testing was performed using custom-manufactured microdilution plates (Merlin Diagnostics, Bornheim, Germany) in accordance to the EUCAST microdilution reference method, and the MICs of amphotericin B, itraconazole, voriconazole, fluconazole, anidulafungin, caspofungin, and micafungin were determined. Candida krusei ATCC 6258 was included in the testing for quality control purposes (20). Plates were read spectrophotometrically at 530 nm.

MICs of amphotericin B, itraconazole, voriconazole, and fluconazole were additionally analyzed using MIC test strips (Liofilchem, Roseto degli, Abruzzi, Italy) on RPMI 1640 agar according to the manufacturer’s recommendations. The essential agreement (EA) between microdilution and MIC test strip was determined. EA was defined as an MIC value within 1 dilution step of the MIC result obtained from the broth microdilution technique. MIC_50_ and MIC_90_ values were determined for both methods, defined as the antifungal concentration at which growth of 50% or 90% of all isolates was inhibited, respectively.

### Morphological characterization of isolates.

To assess morphological differences between *M. capitatus* and *M. clavatus* that could be useful for identification, all isolates were streaked onto the following seven different agars and incubated for 1 to 5 days at 35°C: Columbia blood agar, chocolate agar, Sabouraud agar, or Difco malt extract agar (all Becton Dickinson, Heidelberg, Germany); ChromAgar Orientation (CHROMagar, Paris, France), inhibitory mold agar (in house); and ChromID CPS agar (bioMérieux).

### Molecular analysis of *M. clavatus* isolates.

*M. clavatus* isolates were further analyzed to assess if they belonged to the same clade as during the outbreak of *M. clavatus* in France 2012. The eight regions, including clade-specific SNPs, were amplified by PCR, as previously described (4). Sequencing was performed at SEGENIC (Eurofins Cochin MWG, France) on an ABI Prism 3730XL DNA analyzer (Applied Biosystems, Courtaboeuf, France). Geneious v.6.1.8 software (Biomatters, Auckland, New Zealand) was used to analyze and edit sequences.
