# Supplementary material for: Bloodstream Infections Caused by Magnusiomyces capitatus and Magnusiomyces clavatus: Epidemiological, Clinical, and Microbiological Features of Two Emerging Yeast Species
Source: Antimicrob Agents Chemother. 2022 Feb 15;66(2):e01834-21. doi: 10.1128/aac.01834-21 (PMC8846490; doi:10.1128/aac.01834-21)
Supplement: Supplemental file 1 — Supplemental material. Download aac.01834-21-s0001.pdf, PDF file, 0.5 MB [file aac.01834-21-s0001.pdf]

Bloodstream infections caused by *Magnusiomyces capitatus* and *Magnusiomyces clavatus:* epidemiological, clinical and microbiological features of two emerging yeast species

Janina Noster, Martin Köppel, Marie Desnos-Olivier, Maria Aigner, Oliver Bader, Karl Dichtl, Stephan Göttig, Andrea Haas, Oliver Kurzai, Arthur B. Pranada, Yvonne Stelzer, Grit Walther and Axel Hamprecht

Supplementary data

Table S1. Quality scores obtained by MALDI BioTyper using the direct transfer method. If quality scores were below 1.7 the measurement was repeated using tube-based extraction (indicated by asterisks).

Isolate	Species (ITS sequencing)	MALDI ID	MALDI score
**Mag1**	*M. clavatus*	*M. clavatus*	2.02
**Mag2**			2.01
**Mag3**			1.92
**Mag4**			1.92
**Mag5**			1.87
**Mag6**			2.13*
**Mag7**			1.81
**Mag8**			2.12*
**Mag9**			2.26*
**Mag10**			1.86
**Mag11**			2.18*
**Mag12**			2.10*
**Mag13**			1.81
**Mag14**			2.13*
**Mag15**			1.79*
**Mag16**			1.74
**Mag17**			1.75
**Mag18**			1.92
**Mag19**			1.84*
**Mag20**			1.85
**Mag21**			1.82
**Mag22**			1.87
**Mag23**			1.80*
**Mag24**			1.91
**Mag25**	*M. capitatus*	*M. capitatus*	2.09
**Mag26**			2.23
**Mag27**			1.98
**Mag28**			1.94
**Mag29**			2.06
**Mag30**			1.81
**Mag31**			2.05
**Mag32**			2.07*
**Mag33**			1.81*
**Mag34**			1.93*

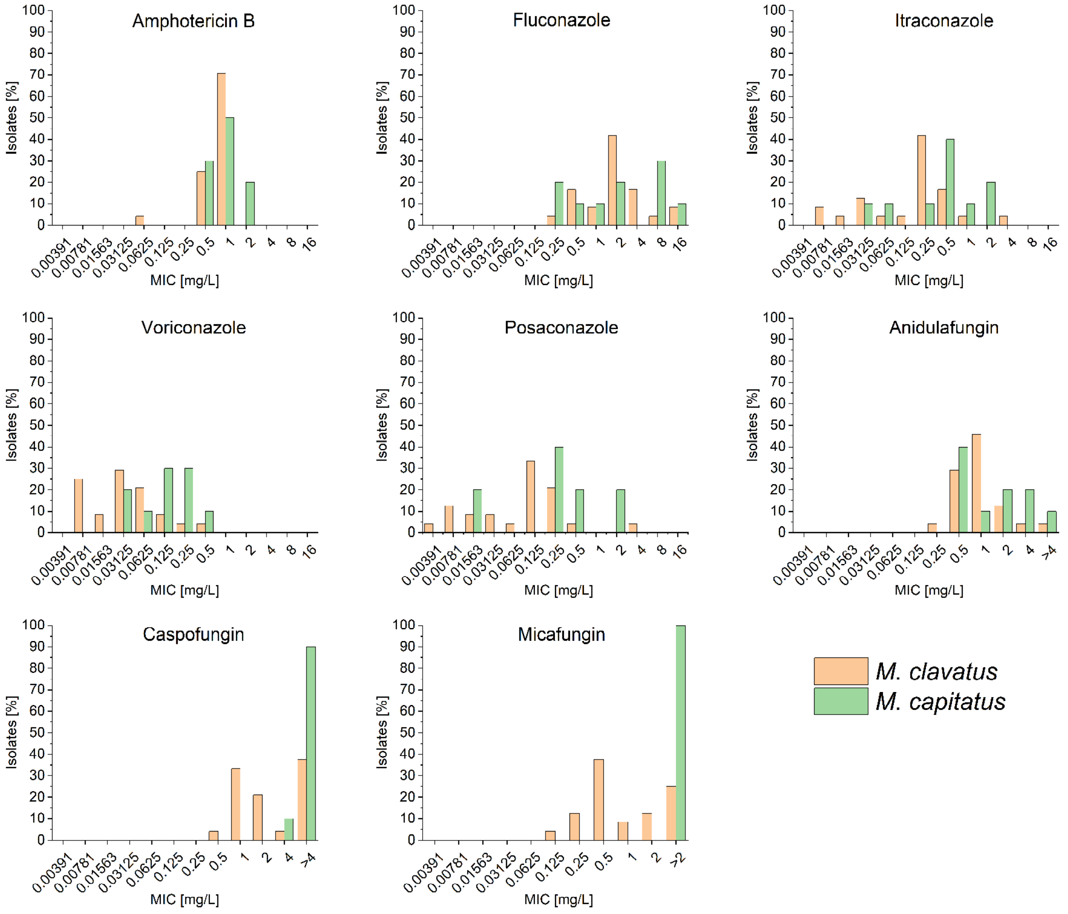

Figure S1. MIC distribution among *Magnusiomyces* isolates. Percentage of isolates of *M. clavatus* and *M. capitatus* with respective MIC values determined using EUCAST BMD method.

Table S2. Antifungal MICs (mg/L) using MIC test strips and broth microdilution (BMD) of *M. clavatus* and *M. capitatus* isolates.

		***M. clavatus* (N = 24)**						***M. capitatus* (N = 10)**					
		**MIC test strips**			**BMD EUCAST**			**MIC test strips**			**BMD EUCAST**		
**Antifungal**	**Reading time [h]**	**MIC range**	**MIC$_{50}$**	**MIC$_{90}$**	**MIC range**	**MIC$_{50}$**	**MIC$_{90}$**	**MIC range**	**MIC$_{50}$**	**MIC$_{90}$**	**MIC range**	**MIC$_{50}$**	**MIC$_{90}$**
**Amphotericin B**	24	0.125-1	0.5	1				0.25-2	1	1			
	48	0.5-2	1	2	0.0625-1	1	1	0.5-2	1	2	0.5-2	1	2
**Itraconazole**	24	0.0078-0.5	0.125	0.25				0.03-4	0.25	2			
	48	0.06-1	0.25	0.5	0.0078-4	0.25	0.5	0.25-32	1	4	0.03-2	0.5	2
**Voriconazole**	24	<0.002-1	0.25	0.5				0.25-1	0.25	1			
	48	0.0156-32	0.5	4	0.0078-0.5	0.03	0.125	0.5-8	1	1	0.03-0.5	0.125	0.25
**Fluconazole**	24	0.25->256	4	32				4-64	8	32			
	48	0.5->256	16	256	0.25-16	2	8	8-64	32	64	0.25-16	2	8

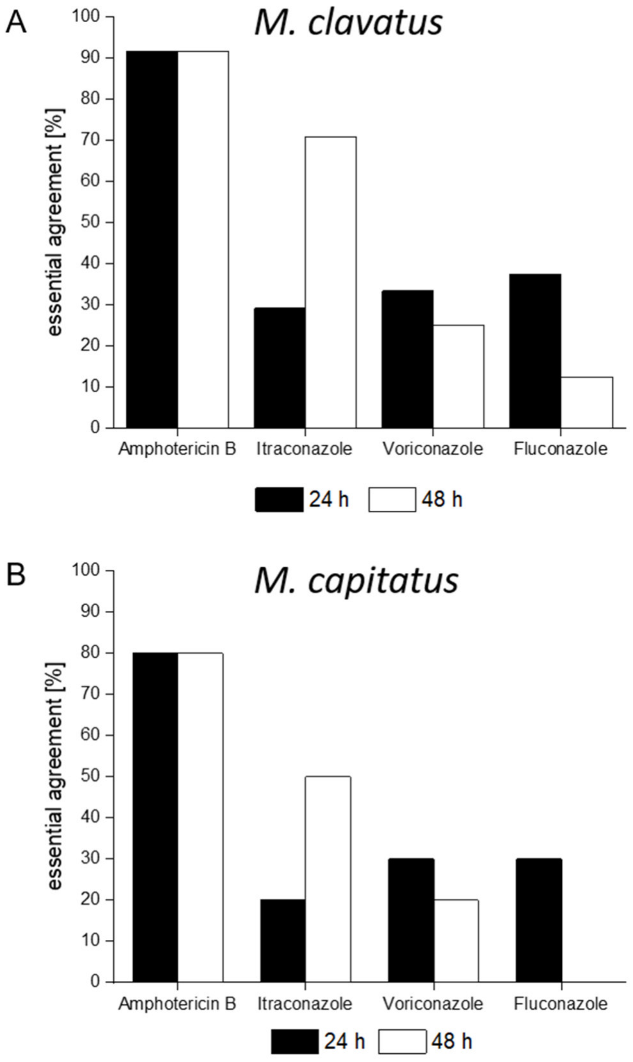

Figure S2. Essential agreement (EA) for susceptibility testing using broth microdilution (reference) and MIC test strips for (A) *M. clavatus* and (B) *M. capitatus* isolates. Incubation for MIC test strip was 24 h or 48 h, respectively. EA was defined as an MIC value within one dilution step of the MIC result obtained by broth microdilution. EA between broth microdilution and MIC test strips read after 24 h is displayed in black and after 48 h in white.

Table S3. Comparison of MICs (mg/L) obtained by MIC test strips and broth microdilution (BMD)

		Microdilution EUCAST – MIC [mg/L]								MIC Test Strip– MIC [mg/L]							
**Strain**	**Isolate**	**AMB**	**FLC**	**ITC**	**VRC**	**POS**	**AFG**	**CAS**	**MFG**	**AMB 24**	**AMB 48**	**ITC 24**	**ITC 48**	**VRC 24**	**VRC 48**	**FLC 24**	**FLC 48**
M. clavatus	Mag1	1	2	0.25	0.03125	0.25	0.5	1	0.5	0.5	1	0.03125	0.25	0.01563	0.25	4	16
	Mag2	1	2	0.5	0.0625	0.25	1	1	0.5	0.5	1	0.125	1	0.25	32	2	>256
	Mag3	1	4	0.25	0.0625	0.125	0.5	1	0.25	0.5	1	0.125	0.5	0.125	0.25	4	16
	Mag4	1	2	0.25	0.0625	0.125	1	2	0.5	1	1	0.25	0.5	0.25	0.5	4	16
	Mag5	1	8	0.125	0.0625	0.0625	>4	>4	>2	1	2	0.0625	0.25	0.0625	0.125	2	4
	Mag6	0.5	16	1	0.25	0.5	1	>4	>2	1	1	0.125	1	1	4	>256	>256
	Mag7	0.5	0.5	0.03125	0.00781	0.03125	1	>4	>2	1	2	0.125	0.25	0.01563	0.01563	2	2
	Mag8	1	4	0.5	0.125	0.125	0.5	1	0.5	1	1	0.125	0.25	0.125	0.25	4	8
	Mag9	0.5	2	0.25	0.03125	0.125	0.5	1	0.25	0.25	1	0.00781	0.125	0.01563	0.125	0.5	8
	Mag10	0.5	2	0.25	0.0625	0.25	1	1	0.5	0.25	1	0.00781	0.125	0.00391	0.0625	0.5	8
	Mag11	1	16	0.5	0.01563	0.125	2	>4	2	0.5	1	0.00781	0.5	0.25	1	4	128
	Mag12	0.5	2	0.25	0.03125	0.25	1	2	0.5	0.125	0.5	0.0625	0.25	<0.0019	0.125	1	8
	Mag13	1	2	0.25	0.03125	0.125	0.5	2	0.125	1	1	0.125	0.5	0.125	0.5	4	8
	Mag14	1	4	0.25	0.03125	0.125	1	1	0.25	0.5	1	0.5	0.5	0.25	0.5	4	32
	Mag15	0.5	0.5	0.5	0.03125	0.01563	2	>4	1	1	1	0.125	0.5	0.5	1	32	64
	Mag16	1	0.5	0.03125	0.00781	0.00781	1	>4	2	0.5	1	0.25	0.25	0.5	1	16	32
	Mag17	1	0.5	0.01563	0.00781	0.01563	0.5	4	2	0.5	1	0.25	0.5	0.25	0.5	16	64
	Mag18	1	1	0.03125	0.00781	0.00781	0.5	1	0.5	1	2	0.0625	0.25	0.25	0.5	32	256
	Mag19	1	2	0.25	0.125	0.125	1	2	>2	1	1	0.0625	0.125	0.25	0.5	8	32
	Mag20	1	0.25	0.00781	0.00781	0.00391	0.25	0.5	0.5	0.5	2	0.125	0.5	0.5	1	32	128
	Mag21	1	2	0.25	0.03125	0.25	4	>4	1	0.5	1	0.25	0.25	0.5	1	32	64
	Mag22	1	1	0.0625	0.01563	0.03125	2	>4	>2	1	1	0.00781	0.0625	0.01563	0.03125	1	1
	Mag23	1	2	0.00781	0.00781	0.00781	1	2	0.5	0.5	0.5	0.125	0.5	0.5	4	8	32
	Mag24	0.0625	4	4	0.5	4	1	>4	>2	0.25	0.5	0.0625	0.125	0.125	0.25	0.25	0.5

M. capitatus	Mag25	2	1	0.0625	0.03125	0.01563	>4	>4	>2	1	1	0.125	0.5	0.25	1	8	32
	Mag26	1	0.25	0.03125	0.03125	0.25	0.5	>4	>2	1	2	2	32	1	8	32	64
	Mag27	1	2	0.5	0.25	2	4	>4	>2	0.25	0.5	0.03125	0.5	0.25	0.5	4	8
	Mag28	1	8	0.5	0.125	0.25	0.5	>4	>2	1	2	4	4	1	1	16	32
	Mag29	0.5	0.25	0.5	0.0625	0.01563	4	>4	>2	0.5	2	0.25	0.25	0.25	0.5	8	32
	Mag30	2	8	2	0.5	2	2	>4	>2	1	1	0.125	1	0.25	1	4	32
	Mag31	1	0.5	2	0.25	0.5	1	>4	>2	0.5	1	0.125	1	0.25	1	16	32
	Mag32	0.5	2	1	0.125	0.5	2	>4	>2	1	1	0.25	0.5	0.5	0.5	8	8
	Mag33	1	8	0.5	0.125	0.25	0.5	>4	>2	1	2	2	2	1	1	32	32
	Mag34	0.5	16	0.25	0.25	0.25	0.5	4	>2	2	2	2	2	1	1	64	64
